# The Effect of the Systemic Inflammatory Response on Plasma Vitamin 25 (OH) D Concentrations Adjusted for Albumin

**DOI:** 10.1371/journal.pone.0092614

**Published:** 2014-03-25

**Authors:** Rawia A. Ghashut, Dinesh Talwar, John Kinsella, Andrew Duncan, Donald C. McMillan

**Affiliations:** 1 Academic Unit of Anaesthesia, College of Medical, Veterinary and Life of Sciences- University of Glasgow, Glasgow Royal Infirmary, Glasgow, United Kingdom; 2 Academic Unit of Surgery, College of Medical, Veterinary and Life of Sciences- University of Glasgow, Glasgow Royal Infirmary, Glasgow, United Kingdom; 3 The Scottish Trace Element and Micronutrient Reference Laboratory, Department of Biochemistry, Glasgow Royal Infirmary, Glasgow, United Kingdom; Oklahoma State University, United States of America

## Abstract

**Background:**

Plasma 25-hydroxyvitamin D (25(OH) D) deficiencies are associated with several diseases. The magnitude of systemic inflammatory response, as evidenced by C-reactive protein (CRP), is a major factor associated with lower 25(OH)D. Other aspects of the systemic inflammatory response may be important in determining plasma 25 (OH)D concentrations.

**Aim:**

To examine the relationship between plasma 25(OH)D, CRP and albumin concentrations in two patient cohorts.

**Methods:**

5327 patients referred for nutritional assessment and 117 patients with critical illness were examined. Plasma 25 (OH) D concentrations were measured using standard methods. Intra and between assay imprecision was <10%.

**Result:**

In the large cohort, plasma 25 (OH) D was significantly associated with CRP (*r*_s_ = −0.113, p<0.001) and albumin (*r*_s_ = 0.192, p<0.001). 3711 patients had CRP concentrations ≤10 mg/L; with decreasing albumin concentrations ≥35, 25–34 and <25 g/l, median concentrations of 25 (OH) D were significantly lower from 35 to 28 to 14 nmol/l (p<0.001). This decrease was significant when albumin concentrations were reduced between 25–34 g/L (p<0.001) and when albumin <25 g/L (p<0.001). 1271 patients had CRP concentrations between 11–80 mg/L; with decreasing albumin concentrations ≥35, 25–34 and <25 g/l, median concentrations of 25 (OH) D were significantly lower from 31 to 24 to 19 nmol/l (p<0.001). This decrease was significant when albumin concentration were 25–34 g/L (p<0.001) and when albumin <25 g/L (p<0.001). 345 patients had CRP concentrations >80 mg/L; with decreasing albumin concentrations ≥35, 25–34 and <25 g/l, median concentrations of 25 (OH) D were not significantly altered varying from 19 to 23 to 23 nmol/l. Similar relationships were also obtained in the cohort of patients with critical illness.

**Conclusion:**

Plasma concentrations of 25(OH) D were independently associated with both CRP and albumin and consistent with the systemic inflammatory response as a major confounding factor in determining vitamin D status.

## Introduction

It has been recognised for some time that plasma 25-hydroxyvitamin D (25(OH)D) deficiencies are associated with increased risks of several diseases such as diabetes, hypertension, stroke, and heart disease [1]. However, there is the potential for confounding factors, since the presence of a systemic inflammatory response has been consistently reported to be associated with low plasma micronutrient concentrations [2]–[4]. In particular, it has recently been reported that the magnitude of systemic inflammatory response, as evidenced CRP, is a major factor associated with the lowering of circulating concentrations of the important lipid soluble vitamins such as 25(OH)D [5]; [6].

Duncan and co-workers [3] reported the presence of an elevated systemic inflammatory response was associated with a significant reduction (41%) in plasma vitamin D concentration when CRP concentrations were >80 mg/L compared to ≤5 mg/L. However, the variability of the association was such that plasma 25(OH)D concentrations were not readily adjusted for CRP concentrations [3].

In the blood 25(OH)D is transported primarily bound to vitamin D binding protein (VDBP) but also to albumin and these fall as part of the systemic inflammatory response [5]; [6]. Albumin is quantitatively the most important protein in the plasma and binds most plasma micronutrients, including 25(OH) D. Moreover, albumin may be considered as a surrogate measure for other plasma binding proteins such as VDBP, and is routinely measured. Therefore, it may be useful in adjusting plasma 25(OH) D concentrations for the effect of the systemic inflammatory response.

The aim of the present study was to examine the relationships between plasma 25(OH) D, CRP and albumin concentrations in a large cohort of patients referred for vitamin D assessment and also examine these relationships in patients with critical illness.

## Patients and Methods

### Nutritional Screen Cohort

A total of 7646 consecutive heparin-treated whole-blood samples from 5327 patients were received from hospitals throughout Scotland between January 2000 and March 2013 for routine analysis of plasma 25 (OH) D concentrations. If more than one set of plasma 25(OH) D results was available in a patient only the first sample was included into the analysis, leaving a total of 5327 plasma 25(OH) D results. As a regional centre blood samples were sent for analysis if the patient was considered at nutritional risk and was often secondary to a number of disease states. In addition, measurement of CRP and albumin was also recorded for these patients.

### Critical Illness Cohort

Patients in the intensive care unit (ICU) of the Royal Infirmary, Glasgow who had respiratory failure requiring ventilatory support, were ≥18 years old, and who had evidence of the systemic inflammatory response syndrome as per Bone’s criteria [7]; [8], and admitted in the period from September 2006 to December 2008 were studied. Briefly, APACHE II score [9] and predicted hospital mortality and SOFA scores, CRP and albumin were recorded. This cohort has been described previously and the purpose of this cohort was to study micronutrients concentrations in the critically ill [10].

This investigation was conducted with the intent of developing local guideline to aid in the interpretation of vitamin 25 (OH) D results. There were two cohorts studied. The first (a large convenience retrospective sample) arose from an audit of patients who had a sample sent to a regional laboratory for a nutritional screen. The second (a small selected prospective cohort) arose from a prospective study of patients with critical illness. The patient data from the two cohorts was anonymized and de-identified prior to analysis (AD and DCM respectively). In line with local ethical procedures written informed consent was obtained for the latter cohort only. The latter study was approved by the ethics committees of the North Glasgow NHS Trust and Multicentre Research Ethics Committee (MREC) Scotland. Where patients were unable to give signed informed consent, consent was obtained from the patients’ next of kin or welfare guardian in accordance with the requirements of the Adults with Incapacity Scotland (2000) Act.

### Analytical Methods

Plasma 25 (OH) D was measured by enzyme immunoassay kit (Immunodiagnostic Systems Ltd) until 2009, when tandem mass spectrometry was used (Waters Acuity; UPLC and TQD). Using routine standards and quality control, intra- and between assay imprecision was <10% in both methods over the concentration range of 25 to 120 nmol/L. Both methods had a similar lower limit of sensitivity of ∼4 nmol/L.

CRP and albumin were measured using an automated analyzer (Architect; Abbot Diagnosis, Maidenhead, UK). The limits of detection for CRP and albumin were 0.2 mg/L and 10 g/L respectively. The interassay CV was <6% over the sample concentration range for albumin and CRP.

### Statistical Analysis

Data was presented in median and (range). Correlations between variables in the control and critically-ill groups were carried out using the Spearman rank correlation. The large cohort was divided into three groups according to CRP concentrations ≤10, 11–80 and >80 mg/L as previously described [11]. The concentrations of individual 25 (OH) D were grouped according to 3 categories of albumin concentrations (≤25, 25–34, ≥35 g/L) as previously described [12]. For each albumin category data are presented as medians and 5th, 10th, 25th, 75th, and 95th percentiles. The concentration of each analyte was compared with the reference albumin category of ≥35 g/L by using the Mann-Whitney *U* test. With each CRP and albumin category the Kruskal-Wallis test was used for the comparison of more than two groups. A P- Value <0.05 was considered significant and the analysis of the data was carried out using SPSS software (version 19; SPSS Inc, Chicago, Ill).

## Results

### Nutritional Screen Cohort

The characteristics of the convenience sample (n = 5327) are shown in Table 1. The majority were older than 50 years (median 58 years), female (67%) and had plasma CRP, albumin and 25 (OH) D in the normal range (median 31 μmol/l). Plasma 25 (OH) D was significantly associated with age (r_s_ = 0.052, p<0.001), sex (males median 29 nmol/l, females median 33 nmol/l, p<0.001), CRP (r_s_ = −0.113, p<0.001) and albumin (r_s_ = 0.192, p<0.001). Age was significantly associated with CRP (r_s_ = 0.275, p<0.001) and albumin (r_s_ = −0.292, p<0.001). CRP was significantly associated with albumin (r_s_ = −0.496, p<0.001).

**Table 1:** Characteristics of nutritional screen cohort and plasma 25 (OH) D concentrations.

	Reference interval	Nutritional screen cohort (n = 5327)
**Age (years)**	NA	58 (16–109)
**Sex (Male/Female)**	NA	1773 (33%)/3554 (67%)
**CRP (mg/l)**	<10	5.0 (0.19–565.0)
**Albumin (g/l)**	35–55	38 (9–52)
**25 (OH) D (nmol/l)**	<25/25–50/>50	31 (6–1140)

**(Median and range).**

**Figure 1:**
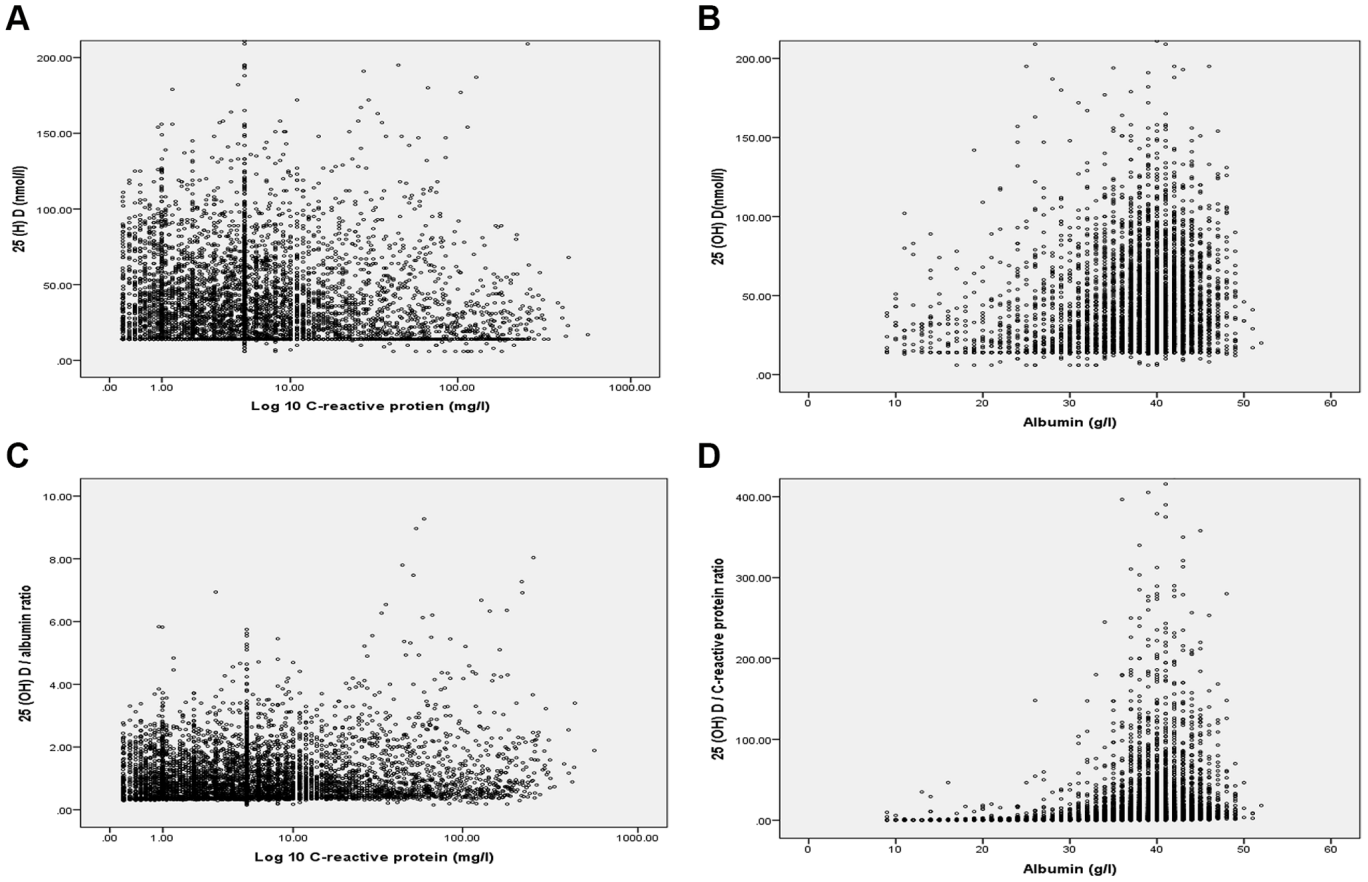
**1a**. The relationship between CRP (log 10) and 25 (OH) D in the nutritional screen cohort (*r_s_* = −0.113, p<0.001). **1b**. The relationship between albumin and 25 (OH) D in the nutritional screen cohort (*r_s_* = 0.192, p<0.001). **1c**. The relationship between CRP (log 10) and 25 (OH) D adjusted to albumin in the nutritional screen cohort (*r_s_* = −0.053, p<0.001). **1d**. The relationship between albumin and 25 (OH) D adjusted to CRP in the nutritional screen cohort (*r_s_* = 0.507, p<0.001).

The association between the magnitude of the systemic inflammatory response, as evidenced by CRP and albumin concentrations, on 25 (OH) D are shown in Figure 1a and 1b. The association of CRP and 25 (OH) D/albumin ratio is shown in Figure 1c; the association of albumin and 25 (OH) D/CRP ratio is shown in Figure 1d.

The median plasma concentrations of 25 (OH) D grouped according to CRP concentrations ≤10, 11–80 and >80 mg/L were 34, 27 and 22 nmol/l respectively (p<0.001) with an overall reduction of 35%. The median plasma concentrations of 25 (OH) D grouped according to albumin concentrations ≥35, 25–34 and <25 g/l were 34, 25 and 20 nmol/l respectively (p<0.001) with an overall reduction of 41%.

The median and the 5th, 10th, 25th, 75th, and 95th percentiles of plasma 25 (OH) D concentrations grouped according to albumin concentrations ≥35, 25–34 and <25 g/l for CRP concentrations ≤10, 11–80 and >80 mg/L are shown in Table 2. 3711 patients had CRP concentrations ≤10 mg/L; with decreasing albumin concentrations ≥35, 25–34 and <25 g/l, median plasma concentrations of 25 (OH) D were significantly lower from 35 to 28 to 14 nmol/l (p<0.001). This decrease was significant when albumin concentrations were between 25–34 g/L (p<0.001) and when albumin concentrations were <25 g/L (p<0.001). 1271 patients had CRP concentrations between 11–80 mg/L; with decreasing albumin concentrations ≥35, 25–34 and <25 g/l, median plasma concentrations of 25 (OH) D were significantly lower from 31 to 24 to 19 nmol/l (p<0.001). This decrease was significant when albumin concentration were 25–34 g/L (p<0.001) and when albumin concentrations were <25 g/L (p<0.001). 345 patients had CRP concentrations >80 mg/L; with decreasing albumin concentrations ≥35, 25–34 and <25 g/l, median plasma concentrations of 25 (OH) D were not significantly altered varying from 19 to 23 to 23 nmol/l.

**Table 2:** Distribution in percentiles of plasma 25 (OH) D according to CRP and albumin concentrations (n = 5327).

CRP concentrations ≤10 mg/L (n = 3711)									
Albumin	Subjects *n* (%)	5th Percentile	10th Percentile	25th Percentile	Median (% change)	75th Percentile	90th Percentile	95th Percentile	*P*-value
**≥35 g/L**	3294(89)	14.0	14.0	20.0	35.0 (100)	57.0	85.0	102.0	<0.001^a^
**25–34** **g/L**	378 (10)	14.0	14.0	15.0	28.0 (80)	46.0	75.0	88.0	<0.001^b^
**<25** **g/L**	39 (1)	14.0	14.0	14.0	14.0 (40)	34.0	64.0	67.0	<0.001^b^
**25 (OH) D/albumin ratio×100**		34.12	36.59	50.00	**85.0**	141.86	210.33	259.68	
**CRP concentrations 11–80 mg/L (n = 1271)**									
**≥35 g/L**	649 (51)	14.0	14.0	18.0	31.0 (100)	52.0	80.0	101.0	<0.001^a^
**25–34** **g/L**	476 (37)	14.0	14.0	14.0	24.0 (77)	49.0	82.0	105.0	<0.001^b^
**<25** **g/L**	146 (11)	14.0	14.0	14.0	19.0 (61)	37.8	76.7	138.5	<0.001^b^
**25 (OH) D/albumin ratio×100**		36.82	39.97	51.81	**84.2**	148.48	242.36	105.71	
**CRP concentrations ≥80 mg/L (n = 345)**									
**≥35 g/L**	42 (12 )	14.0	14.0	14.0	19.0 (100)	26.5	52.6	72.5	0.284^a^
**25–34** **g/L**	150 (43)	10.6	14.0	14.0	23.0 (121)	38.0	58.8	82.8	0.277^b^
**<25** **g/L**	153 (44)	14.0	14.0	14.0	23.0 (121)	33.0	55.2	78.3	0.093^b^
**25 (OH) D/albumin ratio×100**		37.81	42.39	56.55	**90.9**	169.23	265.24	422.29	

a Kruskal Wallis,

b Mann-Whitney *U* test. When n<5 median value was not calculated.

When albumin concentrations were ≥35 g/L, The median plasma concentrations of 25 (OH) D grouped according to CRP concentrations ≤10, 11–80 and >80 mg/L were 35, 31 and 19.0 nmol/l (p<0.001) with an overall reduction of 46%. When albumin concentrations were 25–34 g/L, The median plasma concentrations of 25 (OH) D grouped according to CRP concentrations ≤10, 11–80 and >80 mg/L were 28, 24 and 23.0 nmol/l (p = 0.013) with an overall reduction of 18%. When albumin concentrations were <25 g/L, The median plasma concentrations of 25 (OH) D grouped according to CRP concentrations ≤10, 11–80 and >80 mg/L were 14, 19.0 and 23.0 nmol/l (p = 0.082). The median 25 (OH) D/albumin ratio×100 for CRP concentrations ≤10, 11–80 and >80 mg/L were 85.0, 84.2 and 90.9 (p = 0.002).

### Patients with Critical Illness

The characteristics of the critically ill cohort (n = 117) are shown in Table 3. The majority were older than 50 years (median 60 years), male (65%), an APACHE II score of 21 and SOFA score of 7 and the associated median predicted mortality was 34%. The majority of patients were surgical (55%) and had a median length of ICU stay of 5 days and median hospital stay of 22 days. The median CRP and albumin concentrations were above and below the normal reference intervals respectively. Median adjusted calcium and median urea and creatinine concentrations were within the normal reference interval. Approximately half had some renal impairment eGFR of <60 ml/min. The median plasma 25(OH) D was 33 nmol/l. Plasma 25 (OH) D was significantly associated with age (r_s_ = 0.052, p<0.001), sex (r_s_ = 0.082, p<0.001), CRP (r_s_ = −0.113, p<0.001), albumin (r_s_ = 0.192, p<0.001). Age was significantly associated with sex (r_s_ = 0.030, p = 0.029), CRP (r_s_ = −0.275, p<0.001) and albumin (r_s_ = −0.292, p<0.001). CRP was significantly associated with sex (r_s_ = −0.040, p = 0.003) albumin (r_s_ = −0.496, p<0.001).

**Table 3:** Characteristics of ICU cohort and plasma vitamin D concentrations.

	Reference interval	Nutritional screen cohort (n = 117)
**Age (years)**	NA	60 (18–100)
**Sex (Male/Female)**	NA	76 (65%)/41 (35%)
**C-reactive protein (mg/l)**	<10	109.0 (1.0–565.0)
**Albumin (g/l)**	35–55	17 (9–45)
**25 (OH) D (nmol/l)**	<25/25–50/>50	33 (13–1140)

**(Median and range).**

**Figure 2:**
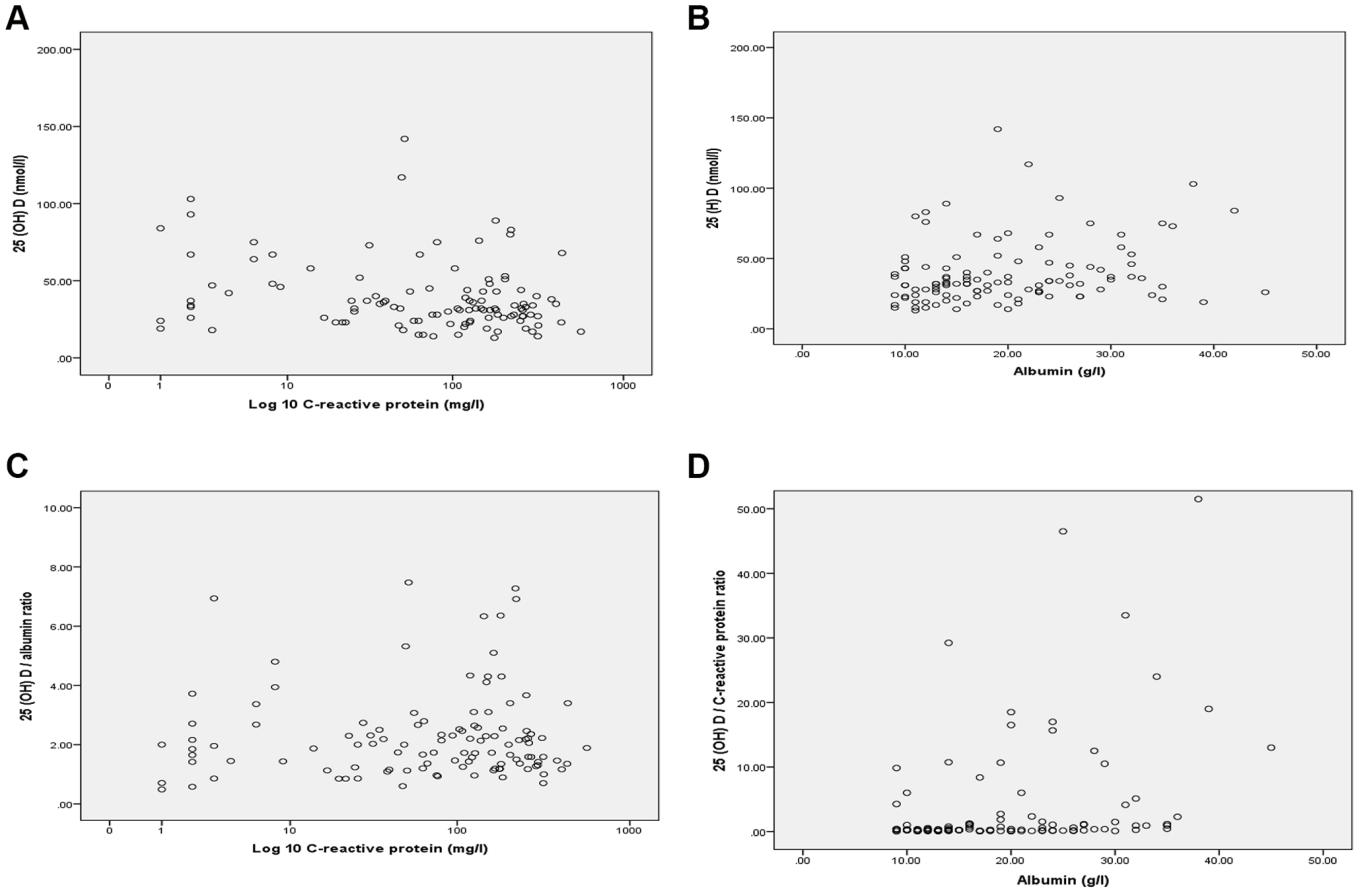
**2a**. The relationship between CRP (log 10) and 25 (OH) D in patients with critical illness (*r_s_* = −0.221, p = 0.017). **2b**. The relationship between albumin and 25 (OH) D in patients with critical illness (*r_s_* = 0.166, p = 0.073). **2c**. The relationship between CRP (log 10) and 25 (OH) D adjusted to albumin in patients with critical illness (*r_s_* = 0.016, p = 0.863). **2d**. The relationship between albumin and 25 (OH) D adjusted to CRP in patients with critical illness (*r_s_* = 0.363, p<0.001).

The association between the magnitude of the systemic inflammatory response, as evidenced by CRP and albumin concentrations, on 25 (OH) D in patients with critical illness are shown in Figure 2a and 2b. The association of CRP and 25 (OH) D/albumin ratio is shown in Figure 2c; the association of albumin and 25 (OH) D/CRP ratio is shown in Figure 2d.

## Discussion

The results of the present study show that, in two different patient cohorts, plasma concentrations of 25(OH) D were associated with the magnitude of the systemic inflammatory response as evidenced by both changes in CRP and albumin concentrations. This effect was differential with CRP most strongly associated with a reduction in plasma 25(OH) D where albumin concentrations were in the normal range (>35 g/l) and with a much weaker association where albumin concentrations were very low (<25 g/l). With normal CRP concentrations (≤10 mg/L), albumin had an independent effect on plasma concentrations of 25(OH) D. These results provide a basis for the better understanding of the impact of the systemic inflammatory response on plasma vitamin D concentrations and for their assessment and interpretation.

There is now wealth of data from observational studies that have shown associations between low concentrations of plasma 25(OH) D and increased risk of cardiovascular disease, cancer, neurodegenerative diseases, disorders of glucose metabolism and death. Many factors such as ageing, ethnicity, season, latitude, adiposity, physical activity, smoking and diet may impact on the link between low 25(OH) D and the above health outcomes. In the present study of patients referred for a nutritional screen some or all of the above factors may have impacted on plasma 25(OH) D concentrations. However, all of the above potential confounding factors appear to be dependent on inflammatory processes for their effect on plasma 25(OH) D concentrations. This is well illustrated in a recent study of the effect of elective knee arthroplasty on plasma 25(OH) D concentrations [5]. Indeed, inflammatory processes have been proposed as a major reason behind the discrepancy in effect of normal vs deficient plasma 25(OH) D concentrations on health outcomes in observational studies (beneficial effect) and interventional (no effect) studies of health outcomes [13]. The similar associations seen in the critical illness cohort in the present study are also consistent with such a concept.

In the present study, the basis of the relatively low median plasma concentrations of 25(OH) D concentrations in those patients with normal C-reactive protein and albumin concentrations is not clear. However, at northerly latitudes such as Scotland plasma concentrations of 25(OH) D concentrations are lower [14]. Indeed, it is recognized that the majority of the Scottish population have plasma 25(OH) D concentrations less than 50 nmol/l considered to be borderline vitamin D status [14]. In such patients with albumin concentrations in the normal range the basis of the inverse relationship between CRP and plasma 25(OH) D might be due to consumption of vitamin D since the concentrations of other binding proteins such as VDBP are also likely to be in the normal range [5]; [6]. Also, there is some evidence that 25(OH)D can be actively taken up by inflammatory cells, particularly macrophages [15]. In contrast, in those patients with low plasma albumin concentrations the lack of such a relationship between CRP and plasma 25(OH) D might be due to loss of vitamin D from the plasma compartment due to redistribution of its binding proteins vitamin D binding protein and albumin [5]. More generally the associations of plasma concentrations of C-reactive, albumin and vitamin D may reflect the redistribution and or consumption of body stores by a chronic ongoing systemic inflammatory response. If this were the case it is likely that similar associations would apply to other micronutrients.

In order to confirm whether the combination of CRP and albumin can better account for the variability of plasma of 25(OH)D it would be important to have access to direct intracellular measurements. For example, red blood cells have been shown to be useful for vitamin B6, vitamin E, B2 zinc and selenium concentrations in subjects undergoing an elective knee arthroplasty with a consequent acute systemic inflammatory response [16]–[18] and in patients with critical illness [10]; [19]–[21]. Whereas plasma concentrations of these vitamins deceased rapidly on activation of the systemic inflammatory response, red blood cell concentrations remained stable. Therefore, alongside the present measurements of plasma 25(OH)D, CRP and albumin concentrations it would be important to examine intracellular concentrations of 25(OH)D to better inform the effect of CRP and albumin on plasma 25(OH)D. Also, this may provide insight into the nature of the effect i.e. whether redistribution or consumption.

In summary, it is now recognized that plasma concentrations of 25(OH)D are confounded in the presence of a systemic inflammatory response, as evidenced by CRP. The results of the present study show that albumin concentration also has an independent effect on plasma concentrations of 25(OH)D. Therefore, plasma concentrations of 25(OH)D may not be reliably interpreted in the presence of abnormal CRP (>10 mg/l) and albumin concentrations (<35 g/l). It may be that plasma concentrations of 25(OH)D in the presence of normal C-reactive and albumin concentrations reliably indicate nutritional status of vitamin D and the need for supplementation.
